# LC-MS-MS Measurements of Urinary Creatinine and the Application of Creatinine Normalization Technique on Cotinine in Smokers' 24 Hour Urine

**DOI:** 10.1155/2012/245415

**Published:** 2012-11-11

**Authors:** Hongwei Hou, Wei Xiong, Xiaotao Zhang, Dongkui Song, Gangling Tang, Qingyuan Hu

**Affiliations:** ^1^China National Tobacco Quality Supervision and Test Center, Zhengzhou 450001, China; ^2^Department of Urology, The First Affiliated Hospital of Zhengzhou University, Zhengzhou 450052, China

## Abstract

A simple and sensitive high performance liquid chromatography-tandem mass spectrometry (HPLC-ESI-MS-MS) method was developed and validated for the quantification of creatinine in human urine. The analysis was carried out on an Agilent Zorbax Eclipse XDB-C18 column (2.1 × 150 mm, 3.5 **μ**m). The mobile phase was 0.1% formic acid in water and 0.1% formic acid in acetonitrile (50/50, v/v). Linear calibration curves were obtained in the concentration range of 1–2000.0 ng/mL, with a lower limit of quantification of 0.99 ng/mL. The intra- and interday precision (RSD) values were below 3%. The method was successfully applied to a bioequivalence study of creatinine in Chinese smokers and nonsmokers. The total cotinine in 24 h urine and cotinine : creatinine ratio were also positively associated (Pearson *R* = 0.942, *P* < 0.0001). However, cotinine : creatinine ratio varied significantly across smoking groups for the difference of individual. 24 h urinary cotinine was more appropriate for expressing correlation with tar than cotinine : creatinine ratio.

## 1. Introduction

There are more than 5000 kinds of chemical substances in cigarette smoke [1]. Exposure to cigarette smoke has been associated with a number of adverse physical effects [2]. Nicotine is a chemical that is present in the mount of about 1% of the weight of cigarette tobacco. Nicotine is the main active ingredient of tobacco and is also the main factor that leads to smoking addiction or dependence producing [3]. Cotinine, a major degradation product of nicotine metabolism, has been an important recognized specific biomarker for evaluating cigarette smoke exposure [4].

Urine specimens are commonly employed in biological monitoring because urine collection is noninvasive and poses minimal infectious disease risk to participants and researchers. Continuous and complete 24 h urine collection yields more accurate results, because spot urine sampling may not provide a valid overview of the entire toxicant exposure profile [5]. Urine sample integrity and completeness is essential to exposure assessment research, and absence of compliance with the collection protocol is a fundamental concern to the researcher. Because creatinine is excreted in urine at a relatively constant rate through glomerular filtration, its measurement is an evaluation of sample integrity and completeness [6, 7]. In addition, urinary creatinine is commonly used in a ratio format to normalize analyte quantification for specimen concentration [8, 9]. The normalization process involves dividing the concentration of the analyte of interest by the creatinine concentration obtained in the same urine sample, with the result reported as the concentration of target analyte per millimol of creatinine. Recently, as a normalization basis, urinary creatinine was used to consider the excretion of a variety of xenobiotics related to smoking, ranging from cotinine to mercapturic acids [10]. 

There are numerous papers published about the determination of creatinine in human fluids, including the Jaffe method [11, 12], enzymatic method [13], flow injection analysis [14], high-performance liquid chromatography [15–22], capillary electrophoretic [23–25], zone electrophoresis [26], gas chromatography-mass spectrometry [27], or liquid chromatography combined with mass spectrometry (LC-MS-MS) [28–30]. Recent determination of creatinine, delta-aminolevulinate, and tyrosine in biological fluids with a direct injection by LC-MS-MS was performed, and isotope dilution tandem mass spectrometry was used to assess the accuracy of creatinine determination in serum, plasma, or mouse plasma [28, 29, 31–33]. Hušková et al. [34] developed a rapid method for the analysis of creatinine in urine by solid-phase extraction MS-MS. Though a time-consuming solid-phase extraction was used for sample preparations, the selectivity of tandem mass spectrometry cannot eliminate all interferences in urine. Another modified LC-MS-MS method was introduced by Park et al. which allowed direct analysing creatinine in 24 h urine after diluted with methanol [30]. However, the analyte was eluted from the column at 0.59 min which cannot be separated from the water dissolved urinary proteins and macromolecules (retention time < 1 min on C18 column). The urinary excretion of creatinine in humans has been a routine test in hospital. The methods based on the color reactions and enzymatic assay are confined by the lack of selectivity. The round robin results revealed considerable and unsatisfying variations between laboratories and methods [35]. 

In order to eliminate the interferences from different instruments for urinary creatinine and use urinary creatinine to normalize smoking related biomarkers in human biological fluids, a sensitive and selective LC-MS-MS method for determining creatinine in urine was developed and verified with enzymatic colorimetric assay. The proposed method was applied to urinary samples from smokers and nonsmokers. The data was applied to adjust cotinine values and was undertaken to explore whether any improvement occurred in the concordance with tar to relate it to tobacco exposure.

## 2. Experimental

### 2.1. Chemicals and Reagents

Acetonitrile, formic acid, and methanol were obtained from TEDIA Company Inc. (OH, USA). All solvents were of HPLC grade. Creatinine was obtained from the United States Pharmacopeial Convention (Rockville, MD, USA). Creatinine-d_3_ (N-methyl-d_3_; purity: 98%; isotopic purity: 99%, Toronto Research Chemicals Inc., Toronto, ON, Canada) was used as internal standard (IS).

### 2.2. Preparation of Stock Solutions, Calibration Standard, and Quality Control Samples

Primary stock solutions of creatinine and creatinine-d_3_ for the preparation of standard and quality control (QC) samples, were prepared by weighing separately. The primary stock solutions (0.21 and 0.1 mg/mL) of the creatinine and creatinine-d_3_, respectively, prepared in water and stored at −80°C were found to be stable for three months (data not shown). Appropriate dilutions were made in water to produce the working stock solutions of 100, 1,000, and 10,000 ng/mL for creatinine for the preparation of calibration curve. Calibrators (1, 2, 5, 10, 20, 50, 200, 500, and 2,000 ng/mL) were freshly prepared by the addition of different aliquots of the working stock solution of the analytes and 25 ng/mL of creatinine-d_3_ to water. Quality control samples for creatinine at three different concentrations (50, 200, and 400 ng/mL) were also prepared with human urine.

### 2.3. Sample Preparation

Frozen urine samples were thawed to room temperature and mixed to suspend any settled precipitate. A 10 *μ*L formic acid was added to 1 mL aliquot of human urine sample, stirred, and centrifuged at 10000 rpm for 10 min. The mixture was filtered through a 0.22 *μ*m polyethersulfone membrane and a 5 *μ*L urine aliquot was transferred to an amber volumetric flask and brought to a total volume of 10 mL with water after being spiked with 100 *μ*L of creatinine-d_3_ internal standard solution (1 *μ*g/mL). A 5 *μ*L aliquot was injected on-column for LC-MS-MS. Another aliquot of urine sample was reserved for enzymatic colorimetric analysis.

The enzymatic colorimetric method was performed in a Hitachi modular automatic analyzer (Roche). Enzymatic method is based on the enzymatic degradation of creatinine and its reaction products by creatininase, creatinase, and sarcosine oxidase. The H_2_O_2_ produced by the oxidation of sarcosine is determined spectrophotometrically.

### 2.4. Instrumental Analysis

All samples were analyzed using an Agilent 1200 liquid chromatograph (Agilent Technologies, Wilmington, DE, USA) coupled with an API 4000 triple quadruple mass spectrometer equipped with a TurboIonSpray source (Applied Biosystems, Foster City, CA, USA). ESI was performed in the positive ion mode (ionspray voltage 4500 V) with nitrogen as nebulizing (gas 1), heater (gas 2), curtain, and collision gas. Gas flow parameters were optimized (nebulizer 40 psi, heater 40 psi and curtain gas 30 psi) by making successive flow injections while introducing mobile phase into the ionization source at 200 *μ*L/min. The declustering potential (73 V), entrance potential (10 eV), collision energy (29 V), and cell exit potential (8 V) were optimized for creatinine by integrated springe pump at a constant flow rate of 10 *μ*L/min. The turbo ion spray temperature was set at 480°C. Quantitative analysis was performed in the multiple reaction monitoring (MRM) mode with a dwell time of 100 ms.

An Agilent Zorbax Eclipse XDB-C18 column (2.1 × 150 mm, 3.5 *μ*m particle size, Agilent Technologies, Wilmington, DE, USA) was used with a flow rate of 200 *μ*L/min at ambient temperature. Isocratic separation was performed with 50% solvent A (0.1% formic acid in water) and 50% solvent B (0.1% formic acid in acetonitrile). Solvents were filtered through a 0.45 *μ*m membrane and degassed by a vacuum before use. Aliquots (5 *μ*L) of the standard or diluted urine samples containing internal standard were injected onto the LC-MS-MS system. The instrument was interfaced to a computer running Applied Biosystems Analyst version 1.5 software.

The enzymatic colorimetric method was performed according to a previously published method [36].

Total urinary cotinine among smokers and nonsmokers was analyzed according to a previously published LC-MS-MS method [37].

### 2.5. Validation Experiments

Several performance parameters were tested to validate the proposed method according to Food and Drug Administration (FDA) guidelines for bioanalytical methods. These were linearity of calibration plots, goodness of fit of calibration plots to the linear regression model, specificity, selectivity, thawing stability, recovery, matrix effects, and precision. 

### 2.6. Urine Samples

The studies were approved by Zhengzhou University Ethics Committee. 246 24 h-urine samples from 82 smokers in three separate days and 57 blank 24 h-urine samples of nonsmokers were obtained at baseline from ongoing studies (Urinary biomarkers related to smoke exposure) in the Institute of Clinical Pharmacology of Zhengzhou University. Informed consent and/or assent were obtained from all of the subjects.

## 3. Results and Discussion

### 3.1. Optimization of the Chromatographic Conditions

During the initial course of method development and validation, several different LC columns and relevant solvent systems were evaluated for the best chromatographic separations of analytes from background interference. Poor peak shapes were observed on most columns, except those with XDB C18 column. The initial mobile phase was chosen as water and methonol (v/v), while poor peak shapes were also observed. In order to get good peak shapes and separation, solvent A (0.1% ammonium acetate in water) and solvent B (0.1% formic acid in methonal) were chosen as mobile phase. 

### 3.2. Mass Chromatograms and Detection Conditions

**Figure 1:**
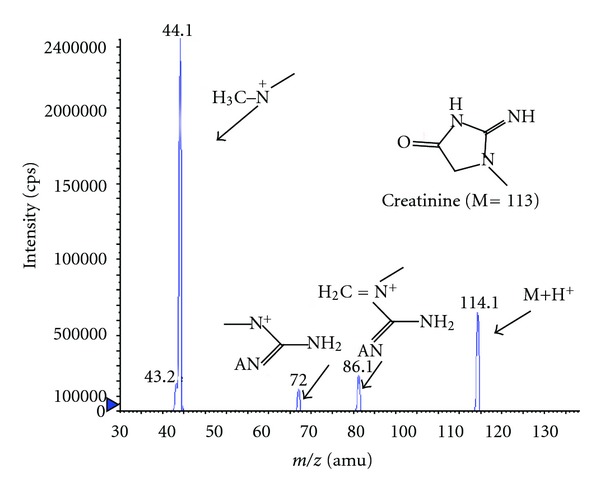
Mass ESI tandem mass spectra of creatinine with product ion scan (*m*/*z* 114).

The detection parameters of MS were optimized using a syringe pump at a flow rate of 10 *μ*L/min. An ESI mass spectrum of creatinine was shown in Figure 1. Under the conditions of ESI, the protonated molecules ([M + H]^+^) of creatinine and creatinine-d_3_ were observed as base peaks at *m*/*z* 114 and 117, respectively. Collision-induced dissociation of both compounds yielded one major fragment ion at *m*/*z* 86 for creatinine and *m*/*z* 89 for creatinine-d_3_, respectively, corresponding to the neutral loss of CO [M + H–CO]^+^. At the same time, the fragment ion [M + H–CO]^+^could yield another main product ion at *m*/*z* 44 for creatinine and *m*/*z* 47 for creatinine-d_3_ (Figure 1). For each analyte, two ion transition pairs were used under multiple reaction mode (MRM). These ion pairs are 114/86 and 114/44 for creatinine for the confirmation and quantification and 117/47 for creatinine-d_3_.

### 3.3. Specificity and Selectivity

**Figure 2:**
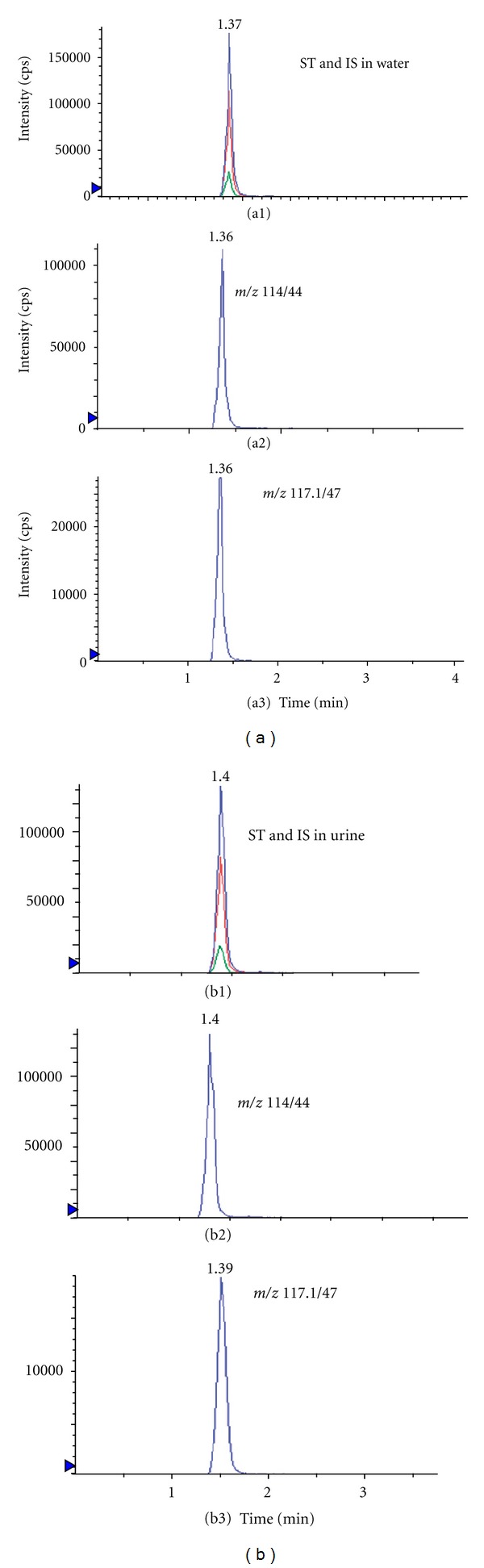
(a) Chromatograms of creatinine standard and creatinine-d_3_, disolved in water (total ion (top), creatinine standard (middle), and creatinine-d_3_ (bottom)). (b) Chromatograms of creatinine and creatinine-d_3_ in 2000-fold diluted urine samples (total ion (top), creatinine standard (middle), and creatinine-d_3_ (bottom)).

No significant interfering peaks from endogenous compounds were observed at the retention times of creatinine and creatinine-d_3_. The retention time of creatinine and creatinine-d_3_ was 1.4 and 1.3 min, respectively. The total chromatographic run time was 12 min. A typical MRM chromatogram of creatinine-d_3_ and creatinine dissolved in water was presented (a1), (a2), and (a3) in Figure 2. Both compounds were detected in a diluted urine sample ((b1), (b2), and (b3) in Figure 2). 

### 3.4. Thawing Stability

Urine matrix underwent a thaw/refreeze cycle during each validation experiment, accumulating six such cycles by the end of the validation. The average values over the six validation experiments for the concentrations of creatinine were 10.09 ± 0.31 mmol/L (CV = 2.1%). The CVs were of similar magnitude to interday CVs, indicating that fast thawing of urine in chilled water did not influence the concentration of analytes.

### 3.5. Matrix Effect and Influence of Dilution

The effect of urine constituents over the ionization of analytes and IS was determined by comparing the responses of the postextracted urine standard QC samples (*n* = 6) with the response of analytes from neat samples at equivalent concentrations. Matrix effect was determined at same concentration of analyte and IS as in recovery experiment.

**Figure 3:**
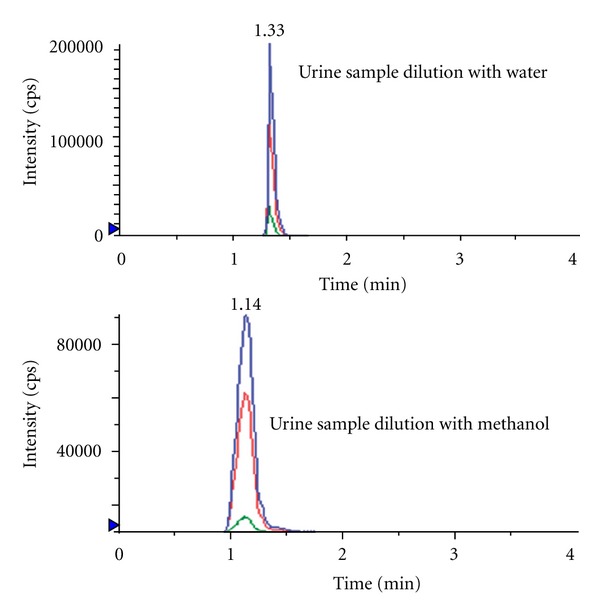
Chromatograms of urine sample (total ion) diluted with water and methanol.

Zinellu et al. [24] and Waterval et al. [33] reported that pretreatment of urine with solid-phase extraction was not a necessary step for urinary creatinine measurement and that simple dilution of urine without pretreatment provided high selectivity for creatinine. On account of this, a simple dilution with water and methanol was applied in this study. As shown in Figure 3, urine samples dilution with water could get clear chromatograms of creatinine. The signal-to-noise ratio in urine diluted with water is higher than with methanol for the narrow creatinine chromatogram peek and low noise. Creatinine was easily detected in all urinary specimens and it also could be better analyzed when the urine samples were diluted to 2000-fold in our assay. In addition, applying a diluted urine sample to the LC-MS-MS could reduce the impact of the matrix effects and thus allow more samples to be processed before cleaning.

### 3.6. Recovery, Calibration Curve, Limits of Detection (LOD) and Quantification (LOQ), and Precisions

Analytical recovery rates were obtained by spiking a nonsmoker pool urine sample with three concentrations of creatinine (50, 200, and 400 ng/mL). Recoveries ranged from 98.6 to 106.0% (*n* = 6) (Table 1).

The peak-area ratios of creatinine to IS were plotted versus creatinine concentration to construct calibration curves. The calibration curve created using creatinine dissolved in water was linear (*y* = 0.0106*x* + 0.00615, *r* = 0.9995) in the analytical range from 1 to 2000 ng/mL. 

LOD and LOQ were determined based on the instrument response with the integrated function of the Analyst 1.5 software (Applied Biosystems). These calculations were based on signal/noise ratios of 3 and 10 for LOD and LOQ, respectively. The corresponding concentrations were calculated from the ratio to the internal standard area on the calibration curve. The LOD and LOQ for creatinine dissolved in water were 0.30 and 0.99 ng/mL.

The intraday precision (RSD) of the methods was established by replicate analyses (*n* = 10) of samples containing low, medium, and high concentration of creatinine. The interday precision (RSD) was established by replicate analyses of the same samples on 10 separate days. Intra-day and interday precisions determined were 1.0–1.8% and 1.5–2.9%, respectively (Table 2). Intra- and interday results showed that the method is reliable.

### 3.7. Comparison of Methods

Published HPLC method, the presence of protein in the injected samples can cause modification of the column end in biased analytical results. Thus, extensive sample cleanups including liquid-liquid (L-L) extraction and SPE were needed to get low sample matrix effects and good HPLC separation for the target compound. However, solid-phase extraction was not a necessary step for urinary creatinine measurement and that simple dilution of the urine sample without pretreatment provided high selectivity for creatinine [38]. In this experiment, a simple dilution with water was used after acid precipitation, centrifugation, and filtration. The creatinine concentration could be well determined and the matrix effect could be extraordinaire light after diluting 2000-fold with water. Compared with published LCMS/MS methods, the new methods required fewer samples (5 *μ*L compared to 50 *μ*L urine) thanks to the lower limit of detection (0.3 ng/mL compared to 22.8 ng/mL [38], 1 ng/mL [30], 6 ng/mL [29], and 3.7 ng/mL [28]). In addition, with the lower limit of detection, this method can be used to measure creatinine concentrations as low as 1 ng/mL. 

**Figure 4:**
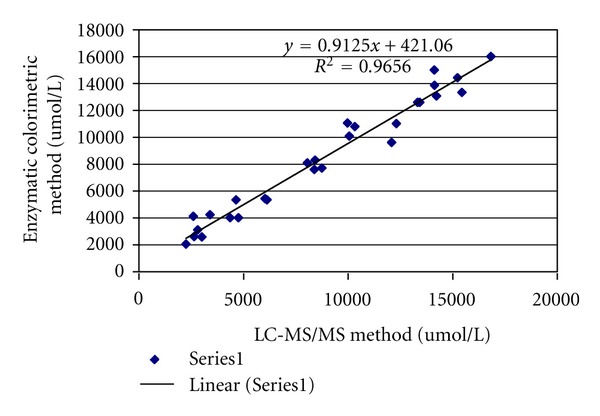
Comparison of human urine creatinine measured by LC-MS-MS and enzymatic colorimetric methods (*n* = 28). Solid line show linear fit lines: = 1.058*x* − 141.486, *R* ^2^ = 0.966, *P* < 0.0001.

To check the effectiveness of LC-MS-MS, 28 24 h-urine samples (16 smokers and 12 nonsmokers) were measured by using the LC-MS-MS with a simple one-step dilution and the enzymatic colorimetric method (for details for enzymatic colorimetric method see Table 1 in supplementary Material available at doi:10.1155/2012/245415) for the same set of urine samples run. The creatinine values measured by the colorimetric and LC-MS-MS methods were positively associated (Pearson *R* = 0.984, *R* ^2^ = 0.968, *P* < 0.0001, Figure 4) (for original data see Table  1 in supplementary material). However, LC-MS-MS has advantage of low detection limits and high selectivity compared with enzymatic colorimetric method. In addition, it can reduce interference. Data from the same instrument could provide more accurate and stable results for creatinine normalization technique.

### 3.8. Effect of Creatinine Normalization Techniques on Cotinine in Smokers' Urine

Cotinine, a major metabolite of nicotine, is the most appropriate parameter to evaluate tobacco exposure and smoking status due to its higher stability and half life when compared to nicotine [9, 39]. Urinary creatinine and cotinine concentrations were determined in 24 h-urine samples (*n* = 246) from 82 smokers and 24 h-urine samples (*n* = 57) from 57 nonsmokers (LC-MS-MS method for the determination of cotinine see supplementary material).

The normalization process involves dividing the concentration of cotinine by the creatinine concentration obtained in the same urine sample, and the result was expressed as the concentration of cotinine per millimol of creatinine. The difference between creatinine normalized and nonnormalized cotinine values and the correlation of total cotinine in 24 h-urine and cotinine creatinine ratio was evaluated. The normalized cotinine values are statistically significantly correlated with nonnormalized concentration (Pearson *R* = 0.858, *P* < 0.0001). Total cotinine in 24 h-urine and cotinine creatinine ratio were also positively associated (Pearson *R* = 0.942, *P* < 0.0001) (Original data see Tables  2–5 in supplementary materia).

**Figure 5:**
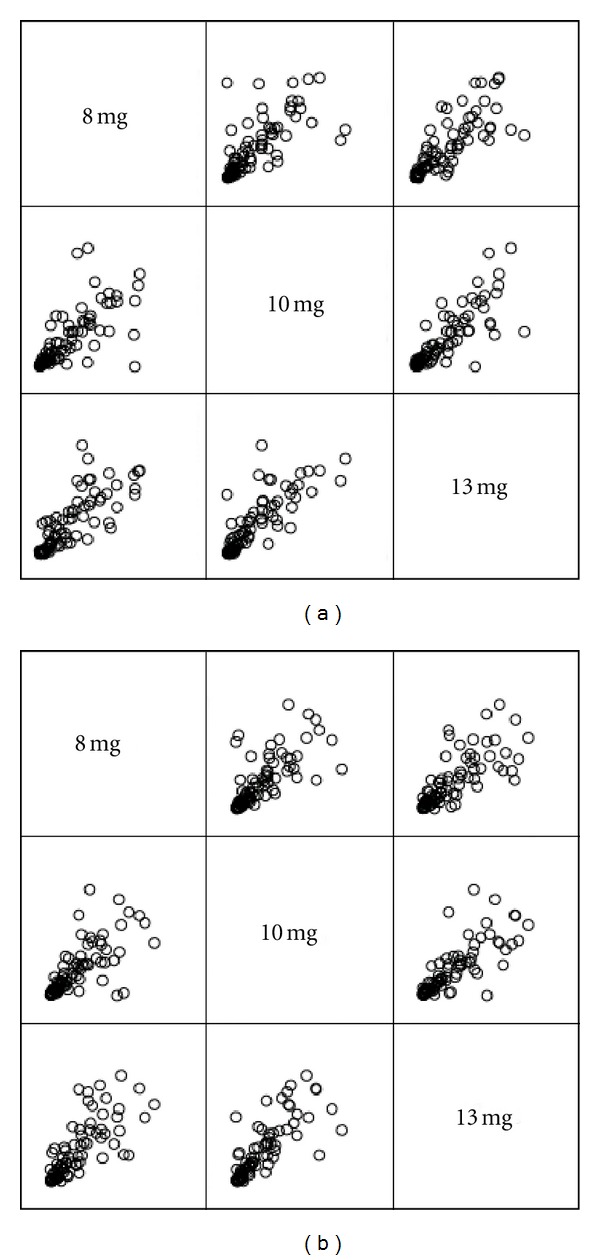
The scatter matrix plot of 24 h urinary cotinine unadjusted (a) and adjusted (b) from smokers with 8 mg, 10 mg, and 13 mg tar yield cigarette.

24 h urine cotinine as a means of assessing exposure to xenobiotics is considered the “gold standard,” which presumably represents the best information on urinary cotinine excretion. 24 h urinary cotinine was positively correlated with tar as in Figure 5(a) (Table 3). Adjusting cotinine values was undertaken to explore whether any improvement occurred in the concordance with tar, and the result was showed in Figure 5(b) (Table 3). It is obvious that the corresponding bubbles in Figure 5(a) were concentrated more than that in Figure 5(b), except 8 mg : 10 mg. There was no use in improving the concordance, except 8 mg : 10 mg (0.716 versus 0.722). The reason may be the influence of age and gender on creatinine production. 

## 4. Conclusions

A simple and specific method was developed for the determination of urinary creatinine by the LC-MS-MS. The sample preparation only involves centrifugation and filtration of diluted urine, which not only allows a high sample throughput but also reduces creatinine background noise and urine salts concentration. In addition, urinary creatinine was used in a ratio format to normalized cotinine concentration.

The normalized cotinine values are statistically significantly correlated with total cotinine in 24 h-urine and cotinine creatinine ratio were also positively associated. Because cotinine : creatinine ratio varied significantly across smoking groups for the difference of individual, 24 h-urinary cotinine was more appropriate for expressing correlation with tar than cotinine : creatinine ratio.

## Supplementary Material

In the Supplementary Material, LC-MS/MS method for cotinine, enzymatic colorimetric method for creatinine, original data for LC-MS-MS and enzymatic colorimetric methods comparison and original data for the application of creatinine normalization technique on cotinine were supported.

## Figures and Tables

**Table 1:** Recovery of the method with dilution of urine samples (2000-fold).

Added (ng/mL)	Detected (ng/mL)	Recovery (*n* = 6, %)
0	223.25	—
50	276.00	105.5
200	435.25	106.0
400	617.75	98.6

**Table 2:** Precision of the method with dilution of urine samples (2000-fold).

Intradays (*n* = 10)		Interdays (*n* = 8)	
Mean (ng/mL)	RSD (%)	Mean (ng/mL)	RSD (%)
335.2	1.0	336.43	2.9
578.4	1.6	575.29	2.0
933.0	1.8	931.29	1.5

**Table 3:** Correlation of 24 h urinary cotinine unadjusted and adjusted with tar.

Variable	*r*		
	8 mg : 10 mg	8 mg : 13 mg	10 mg : 13 mg
24 h cotinine concentration	0.716	0.808	0.822
Cotinine creatinine ratio	0.722	0.765	0.791

## References

[1] Layten Davis D, Nielsen MT (1999). *Tobacco: Production, Chemistry and Technology*.

[2] Repace JL, Lowrey AH (1980). Indoor air pollution, tobacco smoke, and public health. *Science*.

[3] Benowitz NL (1988). Pharmacologic aspects of cigarette smoking and nicotine addiction. *The New England Journal of Medicine*.

[4] Jarvis MJ (1989). Application of biochemical intake markers to passive smoking measurement and risk estimation. *Mutation Research*.

[5] Waikar SS, Sabbisetti VS, Bonventre JV (2010). Normalization of urinary biomarkers to creatinine during changes in glomerular filtration rate. *Kidney International*.

[6] Shaffer PA (1908). The excretion of kreatin and kreatinine in health and disease. *American Journal of Physiology*.

[7] Knudsen N, Christiansen E, Brandt-Christensen M, Nygaard B, Perrild H (2000). Age- and sex-adjusted iodine/creatinine ratio. A new standard in epidemiological surveys? Evaluation of three different estimates of iodine excretion based on casual urine samples and comparison to 24 h values. *European Journal of Clinical Nutrition*.

[8] Bowers LD, Wong ET (1980). Kinetic serum creatinine assays. II. A critical evaluation and review. *Clinical Chemistry*.

[9] Benowitz NL, Dains KM, Dempsey D, Yu L, Jacob P (2010). Estimation of nicotine dose after low-level exposure using plasma and urine nicotine metabolites. *Cancer Epidemiology Biomarkers & Prevention*.

[10] Heavner DL, Morgan WT, Sears SB, Richardson JD, Byrd GD, Ogden MW (2006). Effect of creatinine and specific gravity normalization techniques on xenobiotic biomarkers in smokers’ spot and 24 h urines. *Journal of Pharmaceutical and Biomedical Analysis*.

[11] Jaffé M (1886). Ueber den Niederschlag welchen Pikrinsaure in normalen Harn erzeugt und uber eine neue Reaction des Kreatinins. *Zeitschrift für physiologische Chemie*.

[12] Bonsnes RW, Taussky HH (1945). On the colorimetric determination of creatinine by the Jaffe reaction. *The Journal of Biological Chemistry*.

[13] Jacobs RM, Lumsden JH, Taylor JA, Grift E (1991). Effects of interferents on the kinetic Jaffe reaction and an enzymatic colorimetric test for serum creatinine concentration determination in cats, cows, dogs and horses. *Canadian Journal of Veterinary Research*.

[14] Yao T, Kotegawa K (2002). Simultaneous flow-injection assay of creatinine and creatine in serum by the combined use of a 16-way switching valve, some specific enzyme reactors and a highly selective hydrogen peroxide electrode. *Analytica Chimica Acta*.

[15] Patel CP, George RC (1981). Liquid chromatographic determination of creatinine in serum and urine. *Analytical Chemistry*.

[16] Tsikas D, Wolf A, Frölich JC (2004). Simplified HPLC method for urinary and circulating creatinine. *Clinical Chemistry*.

[17] Hewavitharana AK, Bruce HL (2003). Simultaneous liquid chromatographic determination of creatinine and pseudouridine in bovine urine and the effect of sample pH on the analysis. *Journal of Agricultural and Food Chemistry*.

[18] Jen JF, Hsiao SL, Liu KH (2002). Simultaneous determination of uric acid and creatinine in urine by an eco-friendly solvent-free high performance liquid chromatographic method. *Talanta*.

[19] Marsilio R, Dall’Amico R, Giordano G (1999). Rapid determination of creatinine in serum and urine by ion-pair high-performance liquid chromatography. *International Journal of Clinical & Laboratory Research*.

[20] Jia L, Gao J, Chen X (1998). The determination of creatinine in human urine by capillary zone electrophoresis with photodiode array detection. *Journal of Liquid Chromatography & Related Technologies*.

[21] Catlin DH, Starcevic B (1991). HPLC method for the assay of creatinine in urine. *Journal of Liquid Chromatography*.

[22] Paroni R, Arcelloni C, Fermo I, Bonini PA (1990). Determination of creatinine in serum and urine by a rapid liquid-chromatographic method. *Clinical Chemistry*.

[23] Shi HL, Ma YQ, Ma YF (1995). A simple and fast method to determine and quantify urinary creatinine. *Analytica Chimica Acta*.

[24] Zinellu A, Sotgia S, Zinellu E, Chessa R, Deiana L, Carru C (2006). Assay for the simultaneous determination of guanidinoacetic acid, creatinine and creatine in plasma and urine by capillary electrophoresis UV-detection. *Journal of Separation Science*.

[25] Ruiz-Jiménez J, Mata-Granados JM, de Castro MDL (2007). On-line automatic SPE-CE coupling for the determination of biological markers in urine. *Electrophoresis*.

[26] Gatti R, Lazzarotto V, de Palo CB (1999). A rapid urine creatinine assay by capillary zone electrophoresis. *Electrophoresis*.

[27] Welch MJ, Cohen A, Hertz HS (1986). Determination of serum creatinine by isotope dilution mass spectrometry as a candidate definitive method. *Analytical Chemistry*.

[28] Felitsyn NM, Henderson GN, James MO, Stacpoole PW (2004). Liquid chromatography-tandem mass spectrometry method for the simultaneous determination of *δ*-ALA, tyrosine and creatinine in biological fluids. *Clinica Chimica Acta*.

[29] Takahashi N, Boysen G, Li F, Li Y, Swenberg JA (2007). Tandem mass spectrometry measurements of creatinine in mouse plasma and urine for determining glomerular filtration rate. *Kidney International*.

[30] Park EK, Watanabe T, Gee SJ, Schenker MB, Hammock BD (2008). Creatinine measurements in 24 h urine by liquid chromatography-tandem mass spectrometry. *Journal of Agricultural and Food Chemistry*.

[31] Lawson N, Lang T, Broughton A, Prinsloo P, Turner C, Marenah C (2002). Creatinine assays: time for action?. *Annals of Clinical Biochemistry*.

[32] Stokes P, O’Connor G (2003). Development of a liquid chromatography-mass spectrometry method for the high-accuracy determination of creatinine in serum. *Journal of Chromatography B*.

[33] Waterval WAH (2008). Simultaneous determination of creatine, creatinine and guanidinoacetate in plasma and urine by stable-isotope dilution UPLC-MS/MS. *The Journal of Inherited Metabolic Disease*.

[34] Hušková R, Chrastina P, Adam T, Schneiderka P (2004). Determination of creatinine in urine by tandem mass spectrometry. *Clinica Chimica Acta*.

[35] Tsikas D, Wolf A, Mitschke A, Gutzki F-M, Will W, Bader M (2010). GC-MS determination of creatinine in human biological fluids as pentafluorobenzyl derivative in clinical studies and biomonitoring: inter-laboratory comparison in urine with Jaffé, HPLC and enzymatic assays. *Journal of Chromatography B*.

[36] Guder WG, Hoffmann GE, Hubbuch A (1986). Multicentre evaluation of an enzymatic method for creatinine determination using a sensitive colour reagent. *Journal of Clinical Chemistry and Clinical Biochemistry*.

[37] Fan Z, Xie F, Xia Q, Wang S, Ding L, Liu H (2008). Simultaneous determination of nicotine and its nine metabolites in human urine by LC-MS-MS. *Chromatographia*.

[38] Hušková R, Chrastina P, Adam T, Schneiderka P (2004). Determination of creatinine in urine by tandem mass spectrometry. *Clinica Chimica Acta*.

[39] Petersen GO, Leite CE, Chatkin JM, Thiesen FV (2010). Cotinine as a biomarker of tobacco exposure: development of a HPLC method and comparison of matrices. *Journal of Separation Science*.

