# Supplementary material for: LC-MS-MS Measurements of Urinary Creatinine and the Application of Creatinine Normalization Technique on Cotinine in Smokers' 24 Hour Urine
Source: J Anal Methods Chem. 2012 Nov 11;2012:245415. doi: 10.1155/2012/245415 (PMC3503330; doi:10.1155/2012/245415)
Supplement: Supplementary file 1 — In the Supplementary Material, LC-MS/MS method for cotinine, enzymatic colorimetric method for creatinine, original data for LC-MS-MS and enzymatic colorimetric methods comparison and original data for the application of creatinine normalization technique on cotinine were supported. [file 245415.f1.doc]

**LC-MS-MS Measurements of Urinary Creatinine and its Application to Creatinine Normalization Technique on Cotinine in Smokers’ 24h-urine**

**Hongwei Hou^1^, Wei Xiong^1^, Xiaotao Zhang^1^, Dongkui Song^2^, Gangling Tang^1^, Qingyuan Hu^1^**

^1^China National Tobacco Quality Supervision & Test Center, Zhengzhou, 450001, People’s Republic of China

^2^Department of Urology, the First Affiliated Hospital, Zhengzhou University, Zhengzhou, 450052, People’s

Republic of China

Correspondence should be addressed to Hongwei Hou and Qingyuan Hu, [houhw@ztri.com](mailto:houhw@ztri.com).cn; huqy@ztri.com.cn

**Surporting Information**

**S-1 LC-MS/MS method for cotinine [1]**

*S-1.1. Sample preparetion.* Frozen urine samples were thawed to room temperature and mixed to suspend any settled precipitate. A 20 μL internal standard was added to 100 μL aliquot of human urine sample and then make to 1 mL with water, stirred, and centrifuged at 10000 rpm for 10 min. The mixture was filtered through a 0.22 μm Polyether Sulfone membrane and a 5 μL aliquot was injected on-column for LC-MS/MS.

*S-1.2. Instrumental Analysis.* All samples were analyzed using Agilent 1200 liquid chromatograph (Agilent Technologies, Wilmington, DE) coupled with an API 4000 triple quadruple mass spectrometer equipped with a TurboIonSprayTM source (Applied Biosystems, Foster City, CA). ESI was performed in the positive ion mode (ionspray voltage 5000V) with nitrogen as nebulizing (gas 1)，heater (gas 2)，curtain，and collision gas．Gas flow parameters were optimized (nebulizer 50 psi，heater 50 psi and curtain gas 30 psi) by making successive flow injections while introducing mobile phase into the ionization source at 200 µL/min．The declustering potential (58 V), entrance potential (10 eV), collision energy (28 V), and cell exit potential (10 V) were optimized for creatinine by integrated springe pump at a constant flow rate of 10 µL/min. The turbo ion spray temperature was set at 500 ℃．Quantitative analysis was performed in the multiple reaction monitoring (MRM) mode with a dwell time of 100 ms. These ion pairs are 177.2/80.1, 177.2/98.0 for cotinine for the confirmation and quantification and 180.2/80.1 for cotinine-d_3_.

An Agilent Zorbax Eclipse XDB-C18 column (2.1×150 mm, 3.5 μm particle size, Agilent Technologies, Wilmington, DE) was used with a flow rate of 200 μL min^-1^ at ambient temperature. Isocratic separation was performed with 50% solvent A (0.1 % acetic acid in water) and 50% solvent B (0.1 % acetic acid in methanol). Solvents were filtered through a 0.22 μm membrane and degassed by a vacuum before use. Aliquots (100 μL) diluted urine samples containing internal standard were injected onto the LC-MS/MS system. Total run time was 8 min, and a linear gradient condition was used as follows (time, % of solvent B): 0-2 min, 30 to 30; 2-5 min, 90; 7-8 min, 30-30; at 0.2 mL/min. The instrument was interfaced to a computer running Applied Biosystems Analyst version 1.5 software.

*S-1.3. Chromatograms of standard in water and urine sample*


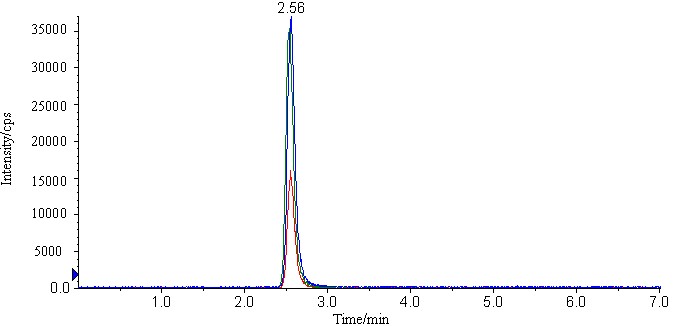


**Fig. 1** Cotinine standard (20 ng/mL) and internal standard in water (40 ng/mL)


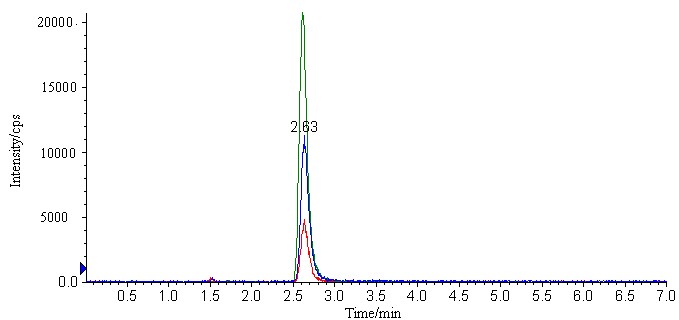


**Fig. 2** Cotinine in a smoker urine (10-fold dilution)

**S-2 Enzymatic colorimetric method for creatinine [2]**

The enzymatic colorimetric method was performed in a Hitachi modular P analyzer (Roche). Enzymatic method is based on the enzymatic degradation of creatinine and its reaction products by creatininase, creatinase and sarcosine oxidase. The H_2_O_2_ produced by the oxidation of sarcosine is determined spectrophotometrically.

*S-2.1. Urine sample dilution.* Urine samples are diluted 1+19 with distilled water, this dilution is taken into account when the results are calculated.

*S-2.2. Materials required.* Calibrator for automated systems, Cat. No. 10759350 190. Controls: Precinorm U plus, Cat.No.12149435 122. 0.9%NaCl

*S-2.3. Reagemts working solutions.* **R1**:TAPS^b^ buffer:30 mmol/L, PH 8.1;creatininase ≥ 333 μkat/L,sarcosone oxidase≥ 133 μkat/L; ascorbate oxidase≥ 33 μkat/L, HTIB:5.9 mmol/L; detergents;presevative. **R2**: TAPS^b^ buffer:50 mmol/L, PH 8.0;creatininase≥ 500 μkat/L; peroxidase≥ 16 μkat/L; 4-aminophenazone:2.0 mmol/L; potassium hexacyanoferrate(Ⅱ):18 μmol/L; detergents;presevative.

General laboratory equipment.

Test principle according to a published method [1].

**LOD** for urine creatinine was 54 μmol/L with the liner range of 30-35360 μmol/L.

**Repeatability:** Within-run and intermediate precision of this method were 0.8% and 2.1%.

**S-3 Original data for LC-MS-MS and enzymatic colorimetric methods comparison**

**Table 1.** Original data from 28 24h-urine samples for methods comparison

| Urine samples | Creatinine for colorimeteric | Creatinine for LC-MS-MS |  | Urine samples | Creatinine for colorimeteric | Creatinine for LC-MS-MS |
| --- | --- | --- | --- | --- | --- | --- |
|  | mg/L | mg/mmoL CRE |  |  | mg/L | mg/mmoL CRE |
| 1 | 15446.00 | 13333.33 |  | 15 | 10065.00 | 10087.72 |
| 2 | 13326.00 | 12587.72 |  | 16 | 4365.00 | 4008.77 |
| 3 | 8749.00 | 7706.14 |  | 17 | 12314.00 | 11008.77 |
| 4 | 12080.00 | 9605.26 |  | 18 | 16831.00 | 16008.77 |
| 5 | 6129.00 | 5337.72 |  | 19 | 4650.00 | 5342.11 |
| 6 | 2609.00 | 4114.04 |  | 20 | 2817.00 | 3114.04 |
| 7 | 3406.00 | 4236.84 |  | 21 | 2649.00 | 2605.26 |
| 8 | 14225.00 | 13070.18 |  | 22 | 15236.00 | 14429.82 |
| 9 | 6039.00 | 5438.60 |  | 23 | 8386.00 | 7605.26 |
| 10 | 3019.00 | 2578.95 |  | 24 | 2255.00 | 2048.25 |
| 11 | 13436.00 | 12587.72 |  | 25 | 9977.00 | 11052.63 |
| 12 | 8427.00 | 8289.47 |  | 26 | 4762.00 | 4013.16 |
| 13 | 10325.00 | 10789.47 |  | 27 | 8061.00 | 8087.72 |
| 14 | 14136.00 | 13859.65 |  | 28 | 14120.00 | 15000.00 |

CRE: creatinin

**S-4 Original data for the application of creatinine normalization technique on cotinine**

The clinical portion of this study was conducted by the First Affiliated Hospital of Zhengzhou University in 2010. A field study with healthy adult subjects aged 20–60 years was performed in Zhengzhou, China in two steps.

Step 1 was run from June to July 2010 and comprised 12 non-smokers and 16 smokers. Urine samples collected in this step were used as method compare (S-3).

Step 2 which served primarily for increasing the number of smokers smoking cigarettes with ISO nicotine yields ranged from 8 to 13 mg/cigarette (Chinese Virginia cigarettes), was run from September to December 2010 and encompassed 82 smokers and 57 nonsmokers as control group. Subjects were recruited by advertisements in regional newspaper and notices at the bulletin-boards of Zhengzhou University. All participating subjects were living in the city of Zhengzhou.

Subjects were excluded if they were under 20 years of age, were pregnant or lactating, participated in any other clinical study within 30 days before study entry, had a history or showed signs of a significant medical or psychiatric condition, used prescription medications within 14 days before study entry, or had a history of alcoholism or drug addiction within a year of study entry, or used alcohol or any nonprescription preparations within 72 h of study entry. A few subjects deviated from the enrollment criteria: underweight, overweight, medication or alcohol usage before study entry, elevated clinical chemistry, abdominal/hernia surgery, and positive drug screen before study entry. Because these deviations were considered minor and not expected to interfere with the study objectives, the subjects were allowed to participate in the study.

Subjects were assigned into one of three tar yield groups, which span the range of tar yields found in commercially available Chinese Virginia cigarettes: 8 mg (low), 10 mg (mid), 13 mg (high). The purpose was to cover a wide range of human nicotine exposure to allow robust correlations between the methodologies.

Subjects were confined to the clinic for six calendar days to give three nonconsecutive periods. In the first two calendar days, the subjects are continued to smoking 15 cigarettes (8 mg) per 24 h, and the second and third calendar days 83 subjects were swtiched to the 10 mg and 13 mg Chinese Virginia cigarettes. 24h-urine samples were collected from each subject for three nonconsecutive days. Collections started at approximately 08:00 hours (first void excluded) and ended at approximately 08;00 hours the following day (first void included). Urine was collected in 2.5 L plastic containers and kept refrigerated throughout the collection period. No chemical preservatives were used. After each 24 h sample collection, volume and pH measurements were recorded, a sample was taken for creatinine analysis, and 20×8 mL and 1×500 mL aliquots were taken and stored frozen at −70 °C until shipped. Aliquots were shipped under dry ice to the analytical laboratory and stored at −80 °C until analysis.

**Table 2.** Original data in 24h-urine samples from 57 nonsmokers

| Sample NO. | Cotinine in urine | Cotinine:  creatinine ratio | Total cotinine in 24h-urine |  | Sample NO. | Cotinine in urine | Cotinine:  creatinine ratio | Total cotinine in 24h-urine |
| --- | --- | --- | --- | --- | --- | --- | --- | --- |
|  | mg/L | mg/mmoL CRE | mg/24h |  |  | mg/L | mg/mmoL CRE | mg/24h |
| 1 | 0.36 | 0.04 | 0.83 |  | 30 | 0.00 | 0.00 | 0.00 |
| 2 | 3.44 | 0.31 | 5.05 |  | 31 | 0.00 | 0.00 | 0.00 |
| 3 | 0.66 | 0.06 | 1.23 |  | 32 | 0.75 | 0.10 | 1.34 |
| 4 | 7.13 | 0.40 | 7.13 |  | 33 | 1.47 | 0.15 | 2.93 |
| 5 | 6.13 | 0.79 | 13.06 |  | 34 | 2.70 | 0.28 | 4.54 |
| 6 | 7.33 | 0.76 | 12.45 |  | 35 | 1.21 | 0.22 | 3.91 |
| 7 | 2.61 | 0.23 | 4.17 |  | 36 | 0.58 | 0.12 | 0.54 |
| 8 | 3.97 | 0.35 | 6.86 |  | 37 | 1.83 | 0.09 | 2.52 |
| 9 | 5.90 | 0.46 | 8.43 |  | 38 | 1.70 | 0.14 | 2.28 |
| 10 | 3.86 | 0.33 | 5.63 |  | 39 | 3.73 | 0.26 | 9.55 |
| 11 | 3.36 | 0.33 | 4.47 |  | 40 | 3.98 | 0.76 | 4.94 |
| 12 | 5.80 | 0.44 | 8.63 |  | 41 | 0.60 | 0.04 | 0.79 |
| 13 | 23.40 | 2.10 | 36.74 |  | 42 | 0.81 | 0.05 | 1.11 |
| 14 | 0.75 | 0.06 | 1.02 |  | 43 | 0.70 | 0.05 | 1.16 |
| 15 | 0.33 | 0.03 | 0.45 |  | 44 | 0.70 | 0.07 | 0.70 |
| 16 | 0.10 | 0.01 | 0.17 |  | 45 | 0.69 | 0.03 | 0.91 |
| 17 | 0.12 | 0.01 | 0.18 |  | 46 | 0.18 | 0.01 | 0.36 |
| 18 | 7.51 | 0.65 | 6.01 |  | 47 | 0.35 | 0.04 | 0.62 |
| 19 | 13.30 | 0.54 | 16.36 |  | 48 | 0.00 | 0.00 | 0.00 |
| 20 | 1.58 | 0.09 | 2.69 |  | 49 | 0.55 | 0.04 | 1.60 |
| 21 | 0.16 | 0.01 | 0.28 |  | 50 | 1.07 | 0.20 | 2.78 |
| 22 | 0.03 | 0.00 | 0.06 |  | 51 | 0.43 | 0.03 | 0.89 |
| 23 | 0.30 | 0.03 | 0.75 |  | 52 | 0.22 | 0.01 | 0.53 |
| 24 | 0.00 | 0.00 | 0.00 |  | 53 | 6.95 | 0.47 | 12.92 |
| 25 | 0.70 | 0.07 | 1.52 |  | 54 | 12.50 | 1.24 | 26.25 |
| 26 | 0.00 | 0.00 | 0.00 |  | 55 | 1.82 | 0.09 | 3.12 |
| 27 | 0.00 | 0.00 | 0.00 |  | 56 | 1.08 | 0.09 | 3.14 |
| 28 | 0.81 | 0.10 | 1.80 |  | 57 | 0.25 | 0.03 | 0.25 |
| 29 | 0.57 | 0.09 | 1.09 |  |  |  |  |  |

**Table 3.** Original data in 82 24h-urine samples from smokers who smoked low tar yield cigarettes (8 mg)

| Sample NO. | Cotinine in urine | Cotinine:  creatinine ratio | Total cotinine in 24h-urine |  | Sample NO. | Cotinine in urine | Cotinine:  creatinine ratio | Total cotinine in 24h-urine |
| --- | --- | --- | --- | --- | --- | --- | --- | --- |
|  | mg/L | mg/mmoL CRE | mg/24h |  |  | mg/L | mg/mmoL CRE | mg/24h |
| L-1 | 1060.00 | 142.13 | 1478.70 |  | L-42 | 1220.00 | 98.56 | 1854.40 |
| L-2 | 27.95 | 3.04 | 51.01 |  | L-43 | 56.50 | 4.16 | 101.14 |
| L-3 | 38.65 | 4.26 | 69.57 |  | L-44 | 401.50 | 30.58 | 634.37 |
| L-4 | 260.00 | 26.98 | 598.00 |  | L-45 | 23.00 | 5.45 | 38.64 |
| L-5 | 127.00 | 22.36 | 167.64 |  | L-46 | 22.50 | 3.89 | 35.10 |
| L-6 | 1760.00 | 182.62 | 2921.60 |  | L-47 | 1595.00 | 200.88 | 2886.95 |
| L-7 | 22.65 | 1.43 | 24.01 |  | L-48 | 1770.00 | 56.07 | 1168.20 |
| L-8 | 47.05 | 8.44 | 63.05 |  | L-49 | 1480.00 | 73.74 | 1568.80 |
| L-9 | 86.70 | 3.17 | 65.89 |  | L-50 | 301.00 | 25.79 | 487.62 |
| L-10 | 1600.00 | 46.76 | 912.00 |  | L-51 | 389.00 | 31.20 | 497.92 |
| L-11 | 28.20 | 2.18 | 43.99 |  | L-52 | 289.50 | 21.97 | 405.30 |
| L-12 | 1505.00 | 108.42 | 1392.13 |  | L-53 | 907.50 | 54.31 | 1134.38 |
| L-13 | 34.55 | 2.67 | 49.75 |  | L-54 | 207.00 | 20.81 | 339.48 |
| L-14 | 184.50 | 26.10 | 361.62 |  | L-55 | 23.70 | 2.85 | 45.98 |
| L-15 | 106.50 | 9.09 | 214.07 |  | L-56 | 1845.00 | 137.28 | 2066.40 |
| L-16 | 506.50 | 96.36 | 1519.50 |  | L-57 | 43.25 | 3.68 | 77.42 |
| L-17 | 540.00 | 74.34 | 1320.30 |  | L-58 | 1245.00 | 172.14 | 3025.35 |
| L-18 | 359.00 | 43.82 | 1005.20 |  | L-59 | 632.50 | 61.67 | 1350.39 |
| L-19 | 227.50 | 27.98 | 534.63 |  | L-60 | 524.00 | 59.65 | 1006.08 |
| L-20 | 474.50 | 58.14 | 1058.14 |  | L-61 | 577.00 | 66.12 | 923.20 |
| L-21 | 51.70 | 5.17 | 111.67 |  | L-62 | 221.50 | 33.20 | 502.81 |
| L-22 | 127.00 | 11.01 | 168.91 |  | L-63 | 692.00 | 61.14 | 1176.40 |
| L-23 | 325.50 | 51.60 | 960.23 |  | L-64 | 297.00 | 42.15 | 794.48 |
| L-24 | 521.50 | 35.00 | 631.02 |  | L-65 | 1375.00 | 132.92 | 3066.25 |
| L-25 | 498.50 | 30.31 | 717.84 |  | L-66 | 357.50 | 14.04 | 284.21 |
| L-26 | 36.40 | 2.26 | 50.60 |  | L-67 | 1495.00 | 76.86 | 1704.30 |
| L-27 | 41.90 | 5.32 | 100.14 |  | L-68 | 811.00 | 151.99 | 2189.70 |
| L-28 | 220.00 | 13.63 | 316.80 |  | L-69 | 181.00 | 12.26 | 181.00 |
| L-29 | 189.00 | 15.89 | 264.60 |  | L-70 | 842.50 | 101.64 | 1685.00 |
| L-30 | 1105.00 | 102.02 | 1303.90 |  | L-71 | 290.00 | 16.86 | 241.57 |
| L-31 | 260.00 | 25.35 | 338.00 |  | L-72 | 110.50 | 14.87 | 85.64 |
| L-32 | 1345.00 | 89.75 | 1566.93 |  | L-73 | 103.15 | 3.23 | 170.20 |
| L-33 | 479.00 | 36.98 | 526.90 |  | L-74 | 1375.00 | 134.06 | 2365.00 |
| L-34 | 91.55 | 6.68 | 128.17 |  | L-75 | 1780.00 | 78.49 | 2136.00 |
| L-35 | 1140.00 | 98.80 | 1892.40 |  | L-76 | 251.00 | 13.68 | 316.26 |
| L-36 | 523.50 | 56.66 | 848.07 |  | L-77 | 496.00 | 57.74 | 684.48 |
| L-37 | 112.50 | 17.98 | 290.25 |  | L-78 | 1285.00 | 81.88 | 1490.60 |
| L-38 | 329.00 | 50.90 | 822.50 |  | L-79 | 99.15 | 7.60 | 114.02 |
| L-39 | 1395.00 | 129.32 | 2915.55 |  | L-80 | 328.00 | 14.96 | 354.24 |
| L-40 | 1090.00 | 123.71 | 2136.40 |  | L-81 | 168.00 | 9.25 | 141.12 |
| L-41 | 1590.00 | 71.65 | 1526.40 |  | L-82 | 1060.00 | 106.09 | 2374.40 |

**Table 4.** Original data in 82 24h-urine samples from smokers who smoked middle tar yield cigarettes (10 mg)

| Sample NO. | Cotinine in urine | Cotinine:  creatinine ratio | Total cotinine in 24h-urine |  | Sample NO. | Cotinine in urine | Cotinine:  creatinine ratio | Total cotinine in 24h-urine |
| --- | --- | --- | --- | --- | --- | --- | --- | --- |
|  | mg/L | mg/mmoL CRE | mg/24h |  |  | mg/L | mg/mmoL CRE | mg/24h |
| M-1 | 100.90 | 4.97 | 131.17 |  | M-42 | 594.50 | 63.91 | 1099.83 |
| M-2 | 49.15 | 4.48 | 84.54 |  | M-43 | 71.30 | 4.93 | 126.20 |
| M-3 | 20.50 | 1.92 | 33.62 |  | M-44 | 334.50 | 30.76 | 715.83 |
| M-4 | 160.00 | 18.26 | 320.00 |  | M-45 | 26.40 | 1.98 | 17.42 |
| M-5 | 100.45 | 14.34 | 106.48 |  | M-46 | 26.35 | 2.59 | 21.34 |
| M-6 | 2225.00 | 139.42 | 2002.50 |  | M-47 | 453.00 | 100.85 | 969.42 |
| M-7 | 28.05 | 1.64 | 26.93 |  | M-48 | 2185.00 | 154.45 | 3452.30 |
| M-8 | 88.90 | 13.42 | 119.13 |  | M-49 | 1415.00 | 98.18 | 1273.50 |
| M-9 | 57.40 | 6.27 | 112.50 |  | M-50 | 161.50 | 24.39 | 218.03 |
| M-10 | 1880.00 | 55.52 | 1259.60 |  | M-51 | 252.50 | 19.36 | 398.95 |
| M-11 | 43.60 | 2.47 | 28.78 |  | M-52 | 282.50 | 28.03 | 452.00 |
| M-12 | 608.00 | 88.22 | 1343.68 |  | M-53 | 628.00 | 53.61 | 653.12 |
| M-13 | 24.30 | 3.81 | 38.76 |  | M-54 | 147.00 | 25.84 | 202.86 |
| M-14 | 777.00 | 57.06 | 1258.74 |  | M-55 | 22.85 | 2.01 | 34.96 |
| M-15 | 129.50 | 35.51 | 424.76 |  | M-56 | 1045.00 | 135.62 | 1933.25 |
| M-16 | 785.50 | 105.39 | 1335.35 |  | M-57 | 43.55 | 3.32 | 65.76 |
| M-17 | 463.00 | 58.67 | 1060.27 |  | M-58 | 1125.00 | 153.39 | 2475.00 |
| M-18 | 264.50 | 42.77 | 814.66 |  | M-59 | 625.50 | 59.95 | 1394.87 |
| M-19 | 286.50 | 42.47 | 733.44 |  | M-60 | 646.00 | 75.95 | 1046.52 |
| M-20 | 466.50 | 49.77 | 1072.95 |  | M-61 | 973.00 | 58.23 | 1050.84 |
| M-21 | 53.50 | 6.56 | 136.43 |  | M-62 | 162.00 | 26.16 | 237.33 |
| M-22 | 90.05 | 8.70 | 161.19 |  | M-63 | 657.50 | 117.11 | 1078.30 |
| M-23 | 250.50 | 38.18 | 571.14 |  | M-64 | 233.50 | 33.03 | 467.00 |
| M-24 | 151.50 | 12.15 | 236.34 |  | M-65 | 1720.00 | 184.39 | 2820.80 |
| M-25 | 866.50 | 75.10 | 1516.38 |  | M-66 | 401.50 | 15.91 | 361.35 |
| M-26 | 19.85 | 2.10 | 45.66 |  | M-67 | 1400.00 | 203.39 | 2576.00 |
| M-27 | 55.45 | 5.57 | 141.40 |  | M-68 | 1045.00 | 159.93 | 1933.25 |
| M-28 | 77.40 | 5.45 | 89.78 |  | M-69 | 117.00 | 12.37 | 226.98 |
| M-29 | 119.50 | 11.22 | 210.32 |  | M-70 | 768.00 | 28.95 | 637.44 |
| M-30 | 924.50 | 117.62 | 1793.53 |  | M-71 | 89.90 | 24.80 | 122.26 |
| M-31 | 557.00 | 35.69 | 704.61 |  | M-72 | 124.00 | 5.80 | 105.40 |
| M-32 | 1285.00 | 71.77 | 1542.00 |  | M-73 | 57.85 | 5.25 | 96.03 |
| M-33 | 800.00 | 71.24 | 1544.00 |  | M-74 | 1445.00 | 83.38 | 2167.50 |
| M-34 | 61.85 | 12.65 | 144.11 |  | M-75 | 1325.00 | 111.42 | 2239.25 |
| M-35 | 1685.00 | 97.73 | 2089.40 |  | M-76 | 550.50 | 26.95 | 561.51 |
| M-36 | 69.55 | 8.60 | 84.85 |  | M-77 | 286.00 | 31.71 | 511.94 |
| M-37 | 78.65 | 10.21 | 165.17 |  | M-78 | 978.00 | 103.47 | 3599.04 |
| M-38 | 336.00 | 61.84 | 661.92 |  | M-79 | 248.50 | 9.15 | 257.20 |
| M-39 | 0.55 | 0.03 | 1.52 |  | M-80 | 323.50 | 10.22 | 355.85 |
| M-40 | 925.50 | 67.31 | 1073.58 |  | M-81 | 161.00 | 10.01 | 207.69 |
| M-41 | 1515.00 | 61.09 | 1454.40 |  | M-82 | 1170.00 | 63.31 | 1977.30 |

**Table 5.** Original data in 82 24h-urine samples from smokers who smoked high tar yield cigarettes (13 mg)

| Sample NO. | Cotinine in urine | Cotinine:  creatinine ratio | Total cotinine in 24h-urine |  | Sample NO. | Cotinine in urine | Cotinine:  creatinine ratio | Total cotinine in 24h-urine |
| --- | --- | --- | --- | --- | --- | --- | --- | --- |
|  | mg/L | mg/mmoL CRE | mg/24h |  |  | mg/L | mg/mmoL CRE | mg/24h |
| H-1 | 297.50 | 52.09 | 465.59 |  | H-42 | 1365.00 | 84.59 | 1638.00 |
| H-2 | 19.90 | 1.67 | 24.28 |  | H-43 | 59.30 | 4.53 | 116.82 |
| H-3 | 21.30 | 1.32 | 33.23 |  | H-44 | 340.00 | 36.28 | 768.40 |
| H-4 | 161.50 | 26.24 | 332.69 |  | H-45 | 39.35 | 4.72 | 44.86 |
| H-5 | 19.95 | 1.46 | 26.83 |  | H-46 | 48.00 | 4.78 | 57.60 |
| H-6 | 1375.00 | 123.42 | 2007.50 |  | H-47 | 1080.00 | 148.42 | 2419.20 |
| H-7 | 90.80 | 24.08 | 114.41 |  | H-48 | 1355.00 | 177.48 | 2249.30 |
| H-8 | 88.60 | 16.97 | 108.09 |  | H-49 | 1530.00 | 172.44 | 2295.00 |
| H-9 | 63.65 | 3.70 | 70.02 |  | H-50 | 539.00 | 23.95 | 528.22 |
| H-10 | 820.50 | 71.11 | 1296.39 |  | H-51 | 399.50 | 36.59 | 587.27 |
| H-11 | 35.25 | 1.73 | 30.32 |  | H-52 | 230.00 | 29.24 | 379.50 |
| H-12 | 1030.00 | 160.46 | 1483.20 |  | H-53 | 511.00 | 48.98 | 572.32 |
| H-13 | 26.25 | 4.06 | 31.50 |  | H-54 | 187.50 | 30.56 | 375.00 |
| H-14 | 143.00 | 17.83 | 327.47 |  | H-55 | 21.75 | 2.47 | 41.33 |
| H-15 | 467.50 | 63.32 | 1014.48 |  | H-56 | 1535.00 | 203.77 | 1857.35 |
| H-16 | 1190.00 | 185.26 | 2284.80 |  | H-57 | 52.40 | 2.71 | 62.36 |
| H-17 | 2215.00 | 155.60 | 3322.50 |  | H-58 | 1320.00 | 179.22 | 2534.40 |
| H-18 | 573.50 | 105.81 | 1284.64 |  | H-59 | 585.50 | 50.36 | 1165.15 |
| H-19 | 384.50 | 66.09 | 1130.43 |  | H-60 | 583.00 | 69.59 | 1107.70 |
| H-20 | 495.00 | 100.33 | 1351.35 |  | H-61 | 955.00 | 75.27 | 1079.15 |
| H-21 | 726.50 | 55.90 | 944.45 |  | H-62 | 198.00 | 13.06 | 245.52 |
| H-22 | 67.20 | 7.98 | 124.99 |  | H-63 | 937.50 | 89.10 | 1603.13 |
| H-23 | 103.40 | 31.69 | 361.90 |  | H-64 | 421.00 | 37.20 | 650.45 |
| H-24 | 165.50 | 10.64 | 256.53 |  | H-65 | 1135.00 | 139.83 | 2553.75 |
| H-25 | 684.00 | 60.20 | 1053.36 |  | H-66 | 602.50 | 24.38 | 602.50 |
| H-26 | 23.00 | 2.60 | 43.24 |  | H-67 | 1240.00 | 99.11 | 1909.60 |
| H-27 | 42.05 | 3.45 | 74.01 |  | H-68 | 382.00 | 50.92 | 809.84 |
| H-28 | 124.00 | 5.83 | 94.24 |  | H-69 | 70.60 | 7.33 | 128.49 |
| H-29 | 125.50 | 8.71 | 129.27 |  | H-70 | 641.50 | 45.49 | 987.91 |
| H-30 | 1375.00 | 129.59 | 2090.00 |  | H-71 | 161.50 | 18.04 | 229.33 |
| H-31 | 720.00 | 46.27 | 1022.40 |  | H-72 | 94.25 | 6.30 | 94.25 |
| H-32 | 1500.00 | 93.47 | 1620.00 |  | H-73 | 50.65 | 3.05 | 97.25 |
| H-33 | 724.00 | 46.93 | 883.28 |  | H-74 | 1655.00 | 97.74 | 2118.40 |
| H-34 | 30.75 | 6.10 | 84.56 |  | H-75 | 1315.00 | 112.67 | 2472.20 |
| H-35 | 1280.00 | 100.19 | 1740.80 |  | H-76 | 718.00 | 33.35 | 703.64 |
| H-36 | 146.50 | 16.87 | 347.21 |  | H-77 | 506.00 | 37.65 | 647.68 |
| H-37 | 80.90 | 10.60 | 186.07 |  | H-78 | 1715.00 | 145.84 | 2915.50 |
| H-38 | 240.50 | 33.75 | 524.29 |  | H-79 | 234.50 | 13.36 | 126.63 |
| H-39 | 1040.00 | 124.40 | 1830.40 |  | H-80 | 262.00 | 22.11 | 298.68 |
| H-40 | 1535.00 | 72.49 | 970.12 |  | H-81 | 51.70 | 7.01 | 49.63 |
| H-41 | 2305.00 | 73.44 | 1500.56 |  | H-82 | 1715.00 | 92.37 | 1440.60 |

**References**

[1] Z. Fan, , F. Xie, Q. L. Xia, S. Wang, L. Ding, and H. M. Liu. 2008. Simultaneous determination of nicotine and its nine metabolites in human urine by LC-MS-MS. Chromatographia. 68 (7-8):623-627. doi: 10.1365/s10337-008-0729-9.

[2] W. G. Guder, G. E. Hoffmann, A. Hubbuch, W. A. Poppe, J. Siedel, C. P. Price. (1986). [Multicentre evaluation of an enzymatic method for creatinine determination using a sensitive colour reagent](http://nbn-resolving.de/urn:nbn:de:kobv:11-100128082). Journal of Clinical Chemistry and Clinical Biochemistry. 24:889-902.
